# Dynamic change of serum CA19–9 levels in benign and malignant patients with obstructive jaundice after biliary drainage and new correction formulas

**DOI:** 10.1186/s12885-021-08204-w

**Published:** 2021-05-07

**Authors:** Bangbo Zhao, Qin Cheng, Hongtao Cao, Xingtong Zhou, Tianhao Li, Liangbo Dong, Weibin Wang

**Affiliations:** 1 Department of General Surgery, State Key Laboratory of Complex Severe and Rare Diseases, Peking Union Medical College Hospital, Chinese Academy of Medical Sciences, Peking Union Medical College, Beijing, China; 2 Department of Brease Surgery, State Key Laboratory of Complex Severe and Rare Diseases, Peking Union Medical College Hospital, Chinese Academy of Medical Sciences, Peking Union Medical College, Beijing, China

**Keywords:** CA19–9, Pancreaticobiliary tumor, Obstructive jaundice, Correction formula

## Abstract

**Background:**

CA19–9 is one of the most widely used tumor markers in biliary-pancreatic diseases. The measured value may not factually reflect the genuine CA19–9 level secreted by tumor, which affected by biliary obstruction. There is an urgent need of developing a correction formula of CA19–9 in biliary obstructive patients to guide clinical practice and avoid making improper clinical decision.

**Methods:**

Clinical characteristics were collected among patients undergoing biliary drainage in our hospital between January 2014 and January 2019. By comparing the malignant and benign patients statistically, dynamic change trend of CA19–9 levels after biliary drainage was obtained. The correction formulas of CA19–9 were generated by means of linear regression.

**Results:**

121 patients, including 102 malignant and 19 benign patients, were enrolled in this study. The baseline CA19–9 level of malignant patients is much higher than that of benign patients. Total bilirubin (TB) level was found to be not related with CA19–9 value (*p* = 0.109). The drop proportion of the average CA19–9 level in the malignant patients (39.2%, IQR -18.4-78.6%) was much lower than that in the benign patients (75.7%, IQR 58.1–86.6%) (*p* = 0.014). The correction formula, CA19–9_True_ = 0.63 × CA19–9_Measured_ - 20.3 (*R*^2^ = 0.693, *p<*0.001), was generated based on the linear relation between CA19–9 after drainage and CA19–9 before drainage in malignant patients, which had similar diagnostic value with true CA19–9 value.

**Conclusions:**

Quantitative correction formulas of CA19–9 considering the effect of biliary decompression was first proposed in this study, aiming to provide a more accurate CA19–9 level to make more accurate clinical decision and avoid making improper therapeutic schedule.

**Supplementary Information:**

The online version contains supplementary material available at 10.1186/s12885-021-08204-w.

## Background

Cancer antigen 19–9 (CA19–9), chemically named monosialoganglioside, was first isolated from a mouse immunized with a colorectal carcinoma cell line [1]. It is a classical tumor marker adopted broadly in biliary and pancreatic diseases to distinguish benign or malignant lesions, hint resectability, evaluate treatment response and estimate prognosis [2]. Out of the above intention, CA19–9 is advised to be measured after neoadjuvant treatment, prior to surgery, following surgery immediately, prior to administration of adjuvant therapy, and for surveillance [3]. Nevertheless, the measuring result may not factually reflect the genuine CA19–9 level secreted by the tumor, which affected by biliary infection, inflammation, biliary obstruction, or other benign conditions, including ovarian cyst, heart failure, hashimoto’s thyroiditis, rheumatoid arthritis and diverticulitis [4–7]. Physiologically, CA19–9 is mainly synthesized by the pancreatic and biliary ductal cells [8]. Elevated pressure of pancreatic and bile duct caused by biliary obstruction might stimulates the secretion of CA19–9, except for the portion secreted by tumors. Therefore, excluding other benign conditions, biliary decompression is advised to be performed in patients with jaundice in order to obtain an accurate baseline with normalized bilirubin, which was first added into the National Comprehensive Cancer Network (NCCN) guidelines for pancreatic adenocarcinoma in version 1.2016 [9].

Through the ages, extremely high level of serum CA19–9 has been considered as a factor of unresectability of pancreatic cancer by surgeons [10–12]. In 2016, the 20th meeting of the International Association of Pancreatology (IAP) sought consensus on a definition of borderline resectable pancreatic ductal adenocarcinoma (BR-PDAC), in which serum CA19–9 level more than 500 U/mL was brought into the biological dimension [13]. Previous studies with regard to CA19–9 and TB mainly focused on proposing a new cutoff value of CA19–9 in differential diagnosis of benign and malignant lesions [4, 5, 14]. Although biliary decompression is advised in the NCCN guideline, there have been no studies focusing on the change scope of serum TB and CA19–9 levels of benign and malignant lesions after biliary decompression so far. In this study, we aimed to evaluate the genuine CA19–9 level in patients with biliary obstruction and propose a correction formula attempting to provide new insight between CA19–9 and TB and help clinicians make more appropriate treatment strategies.

## Methods

### Participants

Between January 2014 and January 2019, 1618 patients underwent endoscopic retrograde cholangiopancreatography (ERCP) or percutaneous transhepaticcholangial drainage (PTCD) to decompress the obstructed common bile duct in Peking Union Medical College Hospital. Patients with confirmed clinical diagnoses verified by radiological or pathological findings were selected. Patients with high serum total bilirubin (>34.2 μmol/L), whose CA19–9 levels were measured before and within 1 month after the decompression operation, were screened. Patients with primary diseases except periampullary and pancreaticobiliary lesions and other possible situations causing elevated serum CA19–9 level, such as teratoma, heart failure, ovarian cyst and hashimoto thyroids, were excluded.

### Data collection

Patients’ demographic information, imaging data, pathological result, date and type of performed operation, diagnosis and serological results (CA19–9, CEA, CA125, liver function, blood routine within 1 week before operation and CA 19–9, total bilirubin within 1 month after operation) were collected. The CA19–9 and total bilirubin were measured by the Laboratory Medicine on standardized platforms (CA19–9, Roche cobas E601; TB, Beckman Coulter® AU 5800).

### Statistical analysis

Numerical variables were expressed by means of median and interquartile range (IQR). The Comparison between serum levels in the 2 groups of patients was obtained by means of the Mann–Whitney U test. Spearman rank correlation was conducted to evaluate the relevance between CA19–9 and other common laboratory variables. The correction formula was obtained by linear regression between CA19–9 and total bilirubin levels. Two-tailed *P* value less than 0.05 was considered statistically significant. All statistical analyses were carried out using IBM SPSS statistics 25.0.

## Results

### Baseline levels of common tumor markers and laboratory indicators in malignant and benign patients with obstructive jaundice

Among the 121 patients enrolled in our study, 102 (84%) patients were found to have malignant diseases and the rest 19 (16%) patients are benign. The sex ratios (male/female) of the malignant and benign groups are 1.27 and 8.5, with the average age of each group are 63 and 60 years.

The serum CA19–9 level of malignant patients is much higher than that of benign patients (malignant group 474.4 (151.8–1325.5) U/mL, benign group 267.7 (141.9–639.9) U/mL) (Table 1). Bile duct cancer caused the highest CA 19–9 level of 571 (158.7–1321.0) U/mL. Pancreatic cancer was the leading cause of high CEA levels, and CA125 levels were higher in other types of cancers, including colorectal cancer and gastric cancer with metastasis. In general, malignant patients have higher CA19–9 (*p* = 0.176), CEA (*p* = 0.074) and CA125 (*p* = 0.486) levels than that of benign patients, however without statistic significance.

**Table 1:** Common tumor markers levels in patients with obstructive jaundice before biliary drainage

Disease	No. (%)	CA19–9 (U/mL)	***P*** value	CEA (ng/mL)	***P*** value	CA125 (U/mL)	***P*** value
Malignant lesions	102	474.4 (151.8–1325.5)	–	3.7 (2.5–7.1)	–	27.6 (12.1–62.0)	–
Pancreatic cancer	44 (43.1%)	494.5 (173.5–1743.3)	–	4.28 (3.0–9.2)	–	43.4 (22.6–163.9)	–
Periampullary cancer	31 (30.4%)	404.2 (79.5–872.0)	0.394^a^	4.5 (3.3–6.3)	0.926	30.2 (12.2–87.8)	0.559
Bile duct cancer	16 (15.7%)	571 (158.7–1321.0)	0.825^a^	3.2 (2.2–5.1)	0.081	11.8 (7.4–14.3)	0.001
Other types of cancers	11 (10.8%)	252 (112.1–595.7)	0.178^a^	2.6 (2.1–9.3)	0.271	50.0 (13.8–121.0)	0.949
Benign lesions	19	267.7 (141.9–639.9)	0.176^b^	2.55 (2.1–4.0)	0.074	10.0 (7.8–232.1)	0.486

^a^ Compared with pancreatic cancer.

^b^ Compare with malignant lesions

To further explore the differences of laboratory results between malignant and benign patients and potential relationship between CA19–9 and these indicators, liver function and blood routine results of malignant and benign patients were collected (Table 2). Red blood cell count (*p* = 0.044) and neutrophil percentage (*p* = 0.016) had statistic differences between the two groups, conforming to the anemia and infection status of cancer patients, with common enzymes related to liver and bile duct having no significant differences between the two groups of patients. In terms of relevance, TB value was found surprisingly to be not related with CA19–9 value (r_s_ = 0.146, *p* = 0.109). CA19–9 value was verified to be more correlated with the severity of biliary infection, with white blood cell count (r_s_ = 0.215, *p* = 0.018) and neutrophil percentage (r_s_ = 0.26, *p* = 0.004) having strong relevance with CA19–9.

**Table 2:** Common laboratory characteristics and their relavance with CA19–9 in patients with malignant and benign lesions

Laboratory characteristics	Malignant	Benign	***P*** value	r_**s**_^**†**^	***P*** value^**†**^
CA19–9, U/mL	474.4 (151.8–1325.5)	267.7 (141.9–639.9)	0.176	–	–
Total bilirubin, μmol/L	207.1 (107.1–340.3)	147.4 (111.3–219.9)	0.148	0.146	0.109
Alanine aminotransferase, U/L	120.5 (51.8–269.8)	135.0 (16.0–198.0)	0.49	0.068	0.456
Aspartate aminotransferase, U/L	543.0 (317.0–959.0)	500.5 (260.8–915.3)	0.805	0.086	0.399
Gamma-glutamyl transpeptidase, U/L	441.0 (284.0–682.3)	418.5 (234.8–603.8)	0.615	0.098	0.342
Alkaline phosphatase, U/L	128.0 (65.0–188.0)	103.0 (58.3–165.8)	0.444	0.066	0.519
Red blood cell count, × 10^12^	3.9 (3.4–4.3)	4.2 (3.6–4.6)	0.044	−0.075	0.416
White blood cell count, ×10^9^	6.4 (5.1–7.9)	5.2 (4.1–8.0)	0.278	0.215	0.018
Neutrophil percentage, %	73.5 (64.5–80.4)	61.5 (55.9–74.6)	0.016	0.26	0.004

†: Relavance with CA19–9 in malignant patients

### Effect of biliary decompression on CA19–9

**Fig. 1:**
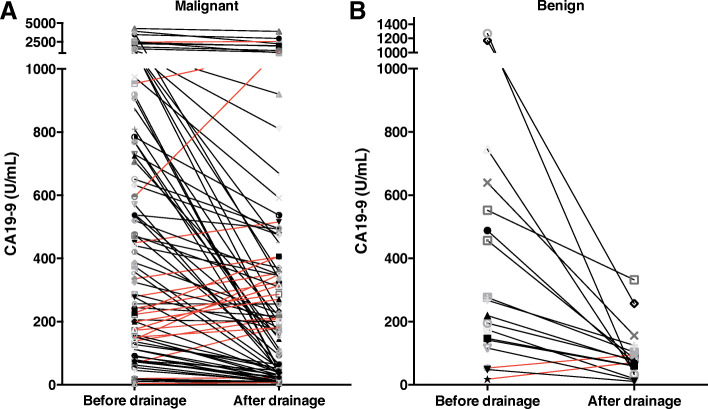
Variation trend of CA19–9 before and after bile duct drainage in malignant (**a**) and benign (**b**) patients with biliary obstruction

After biliary decompression, the variation trend of CA19–9 in benign and malignant patients was portrayed intuitively on basis of the CA19–9 value before and after PTCD or ERCP in malignant and benign patients (Fig. 1). The CA19–9 levels of most patients in the benign group were on a declining curve, and in contrast, the CA19–9 levels had no significant declining trend or even went up in a considerable portion of the malignant patients.

In a quantified view, the drop ratio of the average TB in both malignant (68.4%, IQR 39.9–80.8%) and benign (74.6%, IQR 33.7–83.9%) was similar (*p* = 0.943), indicating the effect of biliary decompression in both groups was the same (Table 3). Nevertheless, the drop proportion of the average CA19–9 level in the malignant patients (39.2%, IQR -18.4-78.6%) was much lower than that in the benign patients (75.7%, IQR 58.1–86.6%) (*p* = 0.014). Not surprisingly, the post-operative average CA19–9 level in the benign patients nearly returned to the normality in 1 week. The difference confirmed the fact that the rise of CA19–9 levels in benign lesions was mainly caused by biliary obstruction, which meant the left CA19–9 in malignant lesions was truly secreted by tumors. The drop ratio of CEA (*p* = 0.306) and CA125 (*p* = 0.051) in the two groups had no obvious differences.

**Table 3:** Drop proportion of CA19–9, CEA, CA125 and total bilirubin after bile duct drainage in patients with malignant and benign lesions

Indicators	Malignant	Benign	***P*** value
CA 19–9	39.2% (−18.4–78.6%)	75.7% (58.1–86.6%)	0.014
CEA	12.6% (−26.2–26.0%)	15.8% (− 2.5–36.3%)	0.306
CA 125	−31.2% (− 196.2–15.8%)	65.5% (31.1–100.0%)	0.051
Total bilirubin	68.4% (39.9–80.8%)	74.6% (33.7–83.9%)	0.943

### Development and assessment of CA19–9 correction formula

With the aim of obtaining the genuine CA19–9 level excluding the affect of biliary obstruction, we tried to fit the pre-operative baseline CA19–9 and TB, and the post-operative CA19–9 or the CA19–9 difference (Table 4). As the abnormal distribution of CA19–9, its absolute and logarithmic values were both used to explore the linear relationship and the samples of CA19–9 more 5000 were excluded for high probability of metastasis. Significant linear relationship was found in the independent and dependent variable couples of CA19–9 before drainage and CA19–9 after drainage (*R*^*2*^ = 0.693, *p<*0.001), log (CA19–9 before drainage) and log (CA19–9 after drainage) (*R*^*2*^ = 0.58, *p<*0.001) in malignant patients. As for benign patients, CA19–9 difference and Log (CA19–9 difference) were found to be linearly related with TB before drainage (*R*^*2*^ = 0.343, *p* = 0.005), CA19–9 before drainage (*R*^*2*^ = 0.950, *p<*0.001) and Log (CA19–9 before drainage) (*R*^*2*^ = 0.669, *p<*0.001) respectively.

**Table 4:** Discovery of linear relationship between CA19–9 or total bilirubin before drainage and CA19–9 after drainage

Independent variable	Dependent variable	Malignant					Benign				
		***R***^***2***^	F	***P*** value	β	α	R^**2**^	F	***P*** value	β	α
Total bilirubin before drainage	CA19–9 after drainage	−0.006	0.459	0.5			−0.047	0.193	0.666		
	Log (CA19–9 after drainage)	0.014	2.274	0.135			−0.035	0.386	0.543		
	CA19–9 difference	−0.011	0.041	0.84			0.343	10.402	0.005	− 2189.017	18.389
	Log (CA19–9 difference)	−0.01	0.057	0.812			−0.036	0.376	0.548		
Log (Total bilirubin before drainage)	Log (CA19–9 after drainage)	0.008	1.751	0.189			−0.055	0.066	0.801		
	Log (CA19–9 difference)	−0.011	0.054	0.816			0.008	1.139	0.301		
CA19–9 before drainage	CA19–9 after drainage	0.693	194.328	<0.001	−20.29	0.632	−0.056	0.05	0.826		
	CA19–9 difference	0.035	3.279	0.073			0.95	306.896	<0.001	−60.412	0.915
Log (CA19–9 before drainage)	Log (CA19–9 after drainage)	0.58	126.904	<0.001	−0.203	0.97	0.073	2.413	0.139		
	Log (CA19–9 difference)	0.019	2.801	0.098			0.669	37.376	<0.001	−2.645	1.887

**Fig. 2:**
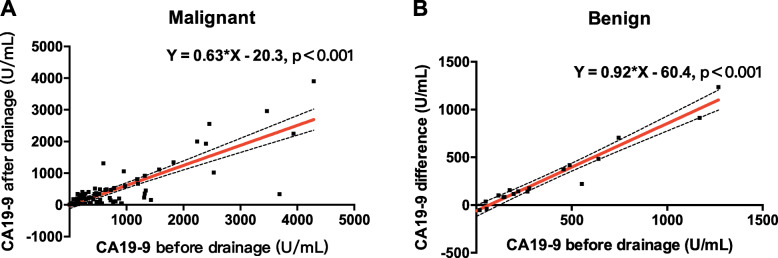
Development of correction formula of CA19–9 in biliary obstructive patients. **a** In malignant lesions, CA19–9 value after biliary drainage had a significant linear relation with CA19–9 value before biliary drainage. **b** In benign lesions, CA19–9 differenece of before and after biliary drainage had a significant linear relation with CA19–9 value before biliary drainage

In malignant patients, a correction formula was generated based on the exploration above that **CA19–9**_**True**_ **= 0.63 × CA19–9**_**Measured**_ **- 20.3** (true CA19–9 value based on CA19–9 value after drainage, measured CA19–9 value based on CA19–9 value before drainage) (Fig. 2a, Supplementary Fig. 1). Corrected by this formula, a biliary obstructive malignant patient with CA19–9 level of 1000 U/mL should have a true CA19–9 level of 612 U/mL. If the true value of CA19–9 was 1000 U/mL, the measured level should be 1614 U/mL. As for benign patients, CA19–9 before drainage strongly CA19–9 difference instead of CA19–9 after drainage, and the formula, CA19–9_Difference_ = 0.92 × CA19–9_Measured_ - 60.4 (measured CA19–9 value based on CA19–9 value before drainage), was obtained (Fig. 2b). We further got a derivation formula **CA19–9**_**True**_ **= 0.085 × CA19–9**_**Measured**_ **+ 60.4**, indicating the limited secretion of CA19–9 by benign lesions.

**Fig. 3:**
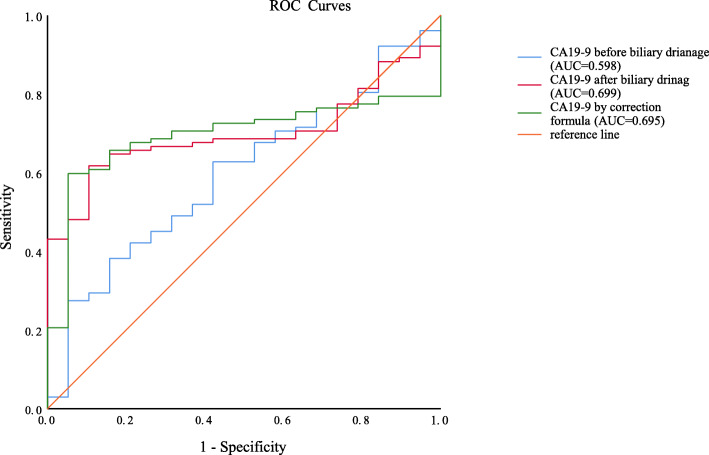
Receiver Operating Characteristic (ROC) curves of CA19–9 before biliary drainage (blue line), CA19–9 after biliary drainage (red line) and CA19–9 by correction formulas (green line) in distinguishing benign and malignant lesions

We then portrayed Receiver Operating Characteristic (ROC) curves of CA19–9 before biliary drainage, CA19–9 after biliary drainage and CA19–9 by correction formulas in distinguishing benign and malignant lesions (Fig. 3). The area under curves (AUC) of CA19–9 before biliary drainage was merely 0.598 (*p* = 0.18) with a dissatisfactory diagnostic value. As for CA19–9 after biliary drainage and CA19–9 by correction formulas, the AUC were 0.699 (*p* = 0.006) and 0.695 (*p* = 0.007) respectively, which indicated that corrected CA19–9 value by our formulas obtained similar diagnostic efficacy with true CA19–9 value.

## Discussion

CA19–9, as a classical tumor marker, play a significant role in the clinical management of pancreaticobiliary tumor since it was first found in 1982 by John L. Magnani [1]. However, the “false positive” and “false negative” in measuring CA19–9 caused by infection, biliary obstruction or Lewis negative imposes restriction on its sensibility and specificity. Hereinto, obstructive jaundice is a broadly recognized factor to make the measured value of CA19–9 higher than the true value. Many malignant patients with biliary obstruction are sentenced to have no opportunity of operation according to the too much high level of CA19–9, which makes some of them lose the chance of cure. Our study first proposed quantitative correction formulas of CA19–9 based on the effect of biliary decompression, aiming to provide a more accurate CA19–9 level to make more accurate clinical decision. Using these formulas, surgeons can have a preliminary estimation of the true value CA19–9 value and tumor load of the patients to avoid making improper therapeutic schedule.

### Probable mechanism of biliary obstruction induced CA 19–9 elevation

The NCCN guidelines for pancreatic adenocarcinoma in version 1.2019 cited two studies to illustrate the relationship between CA19–9 and biliary obstruction. D.V. Mann collected 31 jaundiced patients with high CA19–9 levels and found that relief of jaundice was associated with a fall in CA19–9 level in all benign cases and in nine of the 15 with malignancy and in benign jaundiced cases, a positive correlation was observed between bilirubin and CA19–9 elevation [4]. D. Marrelli obtained the similar results and additionally drew the conclusion that a cut-off value of 90 U/mL to be associated with improved diagnostic accuracy after biliary drainage (sensitivity 61%, specificity 95%) [5]. The previous correction formulas like CA19–9/bilirubin and CA19–9/C-reactive protein (CRP) were restricted to the static value before or after biliary decompression ignoring the dynamic effect [15].

Even though the relationship between CA19–9 and biliary obstruction has been found for nearly 20 years, the internal mechanism that why and how obstructed bile duct can raise the CA19–9 value is still vague. Some hypotheses mainly concentrated on the carrier of CA19–9 from local to the circulation. Mucin4 (MUC4), MUC5AC and bile globular membrane (BGM) are highly specific tumor-associated proteins, which are two carrier proteins of CA19–9 in bile tract [16, 17]. The expression of CA19–9, which was not secreted into the serum, could be observed in normal bile juice and the pancreatic ducts [18]. The increase of CA19–9 caused by biliary obstruction suggested that the high levels of CA19–9 in malignant patients are dependent on an increase of its production as well as an abnormal secretion system [17].

Notably and recently, D.D.Engle and colleagues found that CA19–9 expression in mice resulted in rapid and severe pancreatitis with hyperactivation of epidermal growth factor receptor (EGFR) signaling [19]. Mechanistically, CA19–9 modification of the matricellular protein fibulin-3 increased its interaction with EGFR, and blockade of fibulin-3, EGFR ligands, or CA19–9 prevented EGFR hyperactivation in organoids. CA19–9 was also found to cooperate with the *KrasG12D* oncogene to produce aggressive pancreatic cancer. The newfound potential role of CA19–9 in the course of carcinogenesis and aggression promotion in pancreatic cancer provided a more forceful explanation of the extremely high level of CA19–9 in malignant patients. Nevertheless, the true machanism and quantitative relation between TB or bile duct pressure and CA19–9 still needed more investigation.

### Resectablity evaluation by adjusting baseline CA19–9

Through the ages, extremely high level of serum CA19–9 has been considered as a factor of unresectability of PDAC by surgeons [10–12]. In 2016, the 20th meeting of the International Association of Pancreatology (IAP) sought consensus on a definition of borderline resectable pancreatic ductal adenocarcinoma (BR-PDAC), in which serum CA19–9 level more than 500 U/mL was brought into the biological dimension [13]. N. Santucci reported that of the 171 patients included, 49 (29%) were deemed resectable and 122 (71%) unresectable. Altogether, 93 patients (54%) had jaundice. The area under the ROC curve for CA19–9 as a predictor of resectability was 0.886 (95%CI 0.832–0.932); in jaundiced patients it was 0.880 (95% CI 0.798–0.934). A cut-off in CA 19e9 at 178 UI/ml yielded 85% sensitivity, 81% specificity and 91% positive predictive value for resectability [20].

The more that preoperative CA19–9 increased, the lower were tumor resectability and survival rates. Resectability and 5-year survival varied from 80 to 38% and from 27 to 0% for CA19–9<37 versus ≥4000 U/mL, respectively. The R0 resection rate was as low as 15% in all patients with CA19–9 levels ≥1000 U/mL. CA19–9 increased with the stage of the disease and was highest in AJCC stage IV. Patients with an early postoperative CA19–9 increase had a dismal prognosis [21]. These previous studies concentrating on the relation between CA19–9 and resectability all had the limitation of making use of measured CA19–9 value without correcting it in consideration of biliary obstruction. In the future, we will carry out prospective study and enroll more patients to test and optimize our correction formulas and evaluate resectablility in a brand new view in virtue of the corrected CA19–9 value.

### CA 19–9, the best tumor marker for pancreatic cancer?

The sensibility and specificity of CA19–9 are both nearly 80% as reported. Considering the above-mentioned confounding factors, we cannot help wondering the question, if CA19–9 is the best tumor marker for biliary and pancreatic tumors. More recently discovered tumor markers, such as exosomes, circulating tumor cells and circulating tumor DNA, are being explored in the management of pancreatic cancer.

Pancreatic cancer cell-derived exosomes play critical roles in tumorigenesis and tumor development, and their numerous differentially expressed and functional contents make exosomes promising screening tools. For example, glypican-1 (GPC1), a membrane-anchored protein overexpressed in several tumor types [22], is re-expressed in pancreatic cancer patients through hypomethylation of its promoter [23]. In a study by Melo et al., GPC1(+) circulating exosomes were detected in all enrolled 190 pancreatic cancer patients, with 100% sensitivity and 100% specificity, and from early stages, indicating that it might be a potent early screening biomarker in pancreatic cancer [24]. In 2017, Yang et al. established a signature comprising 5 EV-based protein markers (EGRF, EPCAM, MUC1, GPC1, and WNT2) that provided higher sensitivity (86%) and specificity (81%) than the existing serum marker CA 19–9 or any single EV marker [25]. Circulating tumor cells (CTCs) and circulating tumor DNA released into blood can be relatively easily obtained from minimally invasive liquid biopsies for serial assays and characterization, thereby providing a unique potential for early diagnosis, forecast of disease prognosis, and monitoring of therapeutic response. The potential role of CTCs as an early diagnostic marker has recently been revealed by Rhim et al. Using GEDI chip, CTCs were captured in three different subject groups (pancreatic cancer patients at all stages, patients with precancerous cystic lesion, such as intraductal papillary mucinous neoplasm (IPMN) or mucinous cystic neoplasm, and cancer-free controls). Interestingly, CTCs were detected in 40% (8/21) of the patients with precancerous lesions and circulating pancreas epithelial cells may precede the detectable tumors. The detection rates of CTCs were 73% (8/11) and 0% (0/19) in pancreatic cancer patients and cancer-free group, respectively [26].

These new-found tumor biomarkers showed promising potential in diagnosis, stage evaluation, therapeutic response evaluation, recurrence monitoring and prognosis prediction. However, the relative low homogeneity of these studies, immature and costly testing methods limit the spread and application of these biomarkers. There remains a giant gap between the rising biomarkers and CA19–9, and therefore, deeper digging of the essence of CA19–9 is still helpful for making clinical decision.

### Limitation and application

Although correction formulas with good diagnostic value were obtained in our study, several limitations still existed objectively. First, the sample size was not big enough which may restricted the accuracy of correction formula. On account of the sample size, the enrolled cases were too few to allow us to obtain a satisfactory regression result in each cancer type. Second, our study was carried out retrospectively and the CA19–9 value after biliary drainage was measured in a similar time quantum after ERCP or PTCD, not the same time point, which would cause bias. In the future, prospective study of large samples will be performed to verify the accuracy of our correction formulas and assess their values in other vital issues of pancreatic caner, such as resectability evaluation. Linear relation of CA19–9 before and after biliary decompression in each cancer type will be calculated as well on basis of the increased sample volume.

## Conclusions

Classic tumor markers like CA19–9 played and will keep playing an important role in managing kinds of neoplasms. Quantitative correction formulas of CA19–9 considering the effect of biliary decompression was first proposed in this study, helping surgeons obtain genuine CA19–9 value secreted by tumors to effectively make accurate clinical decision such as resectablity evaluation. Using these formulas, surgeons can have a preliminary estimation of the true value CA19–9 value and tumor load of the patients to avoid making improper therapeutic schedule.

## Supplementary Information

**Additional file 1: Supplementary Fig. 1** Linear regression of CA19–9 before and after biliary drainage in each cancer type. (A) Pancreatic cancer. (B) Periampullary cancer. (C) Bile duct cancer. (D) Other types of cancer.

## Data Availability

The raw data can be requested from the corresponding author for reasonable purpose.

## Abbreviations

CA19–9: Carbohydrate antigen 19–9
TB: Total bilirubin
IQR: Interquartile range
NCCN: National Comprehensive Cancer Network
IAP: International Association of Pancreatology
BR-PDAC: Borderline resectable pancreatic ductal adenocarcinoma
ERCP: Endoscopic retrograde cholangiopancreatography
PTCD: Percutaneous transhepatic cholangial drainage
CEA: Carcinoma embryonic antigen
CA125: Carbohydrate antigen 125
ROC: Receiver operating characteristic
AUC: Area under the curve
MUC: Mucin
EGFR: Epidermal growth factor receptor
GPC1: Glypican-1
EPCAM: Epithelial cell adhesion molecule
CTCs: Circulating tumor cells
IPMN: Intraductal papillary mucinous neoplasm
