# Supplementary figures and images for: Dynamic change of serum CA19–9 levels in benign and malignant patients with obstructive jaundice after biliary drainage and new correction formulas
Source: BMC Cancer. 2021 May 7;21:517. doi: 10.1186/s12885-021-08204-w (PMC8105938; doi:10.1186/s12885-021-08204-w)

**Pancreatic cancer (n=44)**

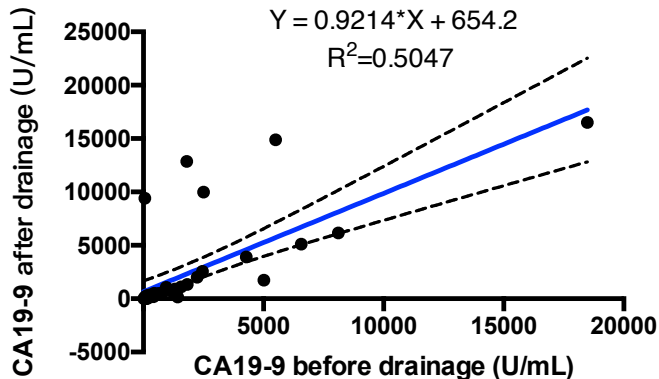

**Periampullary cancer (n=16)**

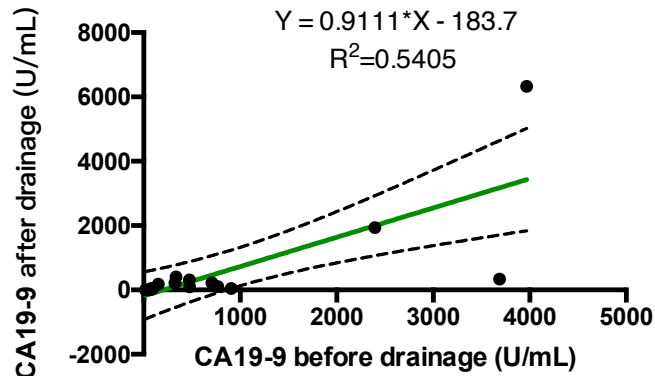

**Bile duct cancer (n=31)**

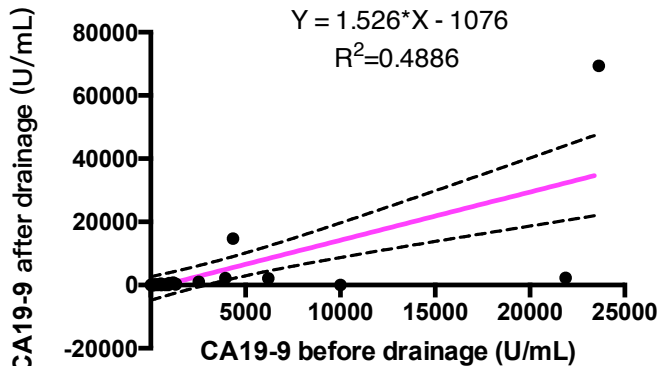

**Other types of cancers (n=11)**

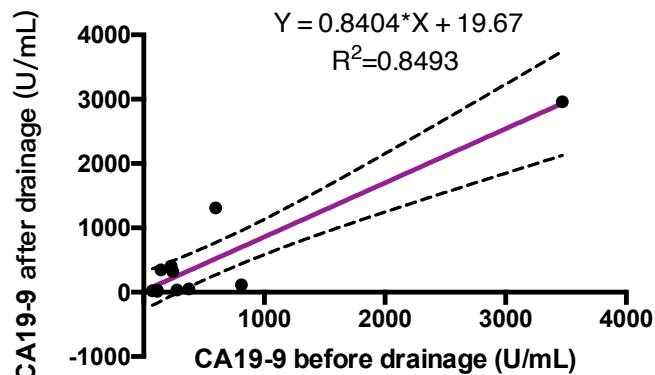

Supplement: Supplementary file 1 — Additional file 1: Supplementary Fig. 1 Linear regression of CA19–9 before and after biliary drainage in each cancer type. (A) Pancreatic cancer. (B) Periampullary cancer. (C) Bile duct cancer. (D) Other types of cancer. [file 12885_2021_8204_MOESM1_ESM.pdf]
